# Leadership Styles and Nurses’ Job Satisfaction. Results of a Systematic Review

**DOI:** 10.3390/ijerph18041552

**Published:** 2021-02-06

**Authors:** Maria Lucia Specchia, Maria Rosaria Cozzolino, Elettra Carini, Andrea Di Pilla, Caterina Galletti, Walter Ricciardi, Gianfranco Damiani

**Affiliations:** 1 Department of Woman and Child Health and Public Health, Fondazione Policlinico Universitario A. Gemelli IRCCS, Via della Pineta Sacchetti, 00168 Rome, Italy; marialucia.specchia@unicatt.it (M.L.S.); walter.ricciardi@unicatt.it (W.R.); gianfranco.damiani@unicatt.it (G.D.); 2 Faculty of Medicine and Surgery, Università Cattolica del Sacro Cuore, Largo Francesco Vito, 00168 Rome, Italy; elettra.carini01@icatt.it (E.C.); andreadipilla.adp@hotmail.com (A.D.P.); caterina.galletti@unicatt.it (C.G.); 3 Emergency Department, Barking, Havering and Redbridge University Hospitals Trust, Rom Valley Way, Romford RM70AG, UK

**Keywords:** leadership, job satisfaction, nursing

## Abstract

Healthcare organisations are social systems in which human resources are the most important factor. Leadership plays a key role, affecting outcomes for professionals, patients and work environment. The aim of this research was to identify and analyse the knowledge present to date concerning the correlation between leadership styles and nurses’ job satisfaction. A systematic review was carried out on PubMed, CINAHL and Embase using the following inclusion criteria: impact of different leadership styles on nurses’ job satisfaction; secondary care; nursing setting; full-text available; English or Italian language. From 11,813 initial titles, 12 studies were selected. Of these, 88% showed a significant correlation between leadership style and nurses’ job satisfaction. Transformational style had the highest number of positive correlations followed by authentic, resonant and servant styles. Passive-avoidant and laissez-faire styles, instead, showed a negative correlation with job satisfaction in all cases. Only the transactional style showed both positive and negative correlation. In this challenging environment, leaders need to promote technical and professional competencies, but also act to improve staff satisfaction and morale. It is necessary to identify and fill the gaps in leadership knowledge as a future objective to positively affect health professionals’ job satisfaction and therefore healthcare quality indicators.

## 1. Introduction

In a constantly challenging environment, healthcare systems are expected to achieve the often competing aims of improving public health, while simultaneously avoiding increases in health spending [1]. Several studies have shown that managers’ approach and leadership styles may influence both staff performance and healthcare system performance metrics [2]. Despite this, questions remain regarding the relationship between leadership styles and variables such as job satisfaction, commitment, and performance among healthcare staff.

In general, leadership is defined as the art of influencing others to achieve their maximum potential to accomplish any task, objective, or project [3]. Leadership theories have historical roots and have developed, presenting different peculiarities over time, as people and historical circumstances have changed [4]. Over the years, different leadership styles have evolved, such as:

*Transformational:* Characterised by charismatic influence, effective communication, valorisation of relationships, and individualised consideration. Leaders know how to convey a sense of loyalty through shared goals, and this results in increased productivity, improved morale and employees’ job satisfaction [5]. Transformational leaders use idealized influence, inspiration and motivation, intellectual stimulation, and individualized consideration to achieve superior results. They motivate others to do more than they originally intended and often more than they thought possible [6]. Transformational leaders work to inspire their followers to look past their own self-interest and to perform above expectations to promote team and organizational interests [7].

*Transactional:* Characterised by processes of recognition, reward or punishment, corrective actions by the leader based on how the employees perform the tasks assigned to them. Staff generally work independently, there is no cooperation between employees who show a commitment to the organisation in the short term [8]. Transactional leadership fails to build trust between the leader and the follower; it does not require a leader to take the ethical and moral road and relies on extrinsically motivating the employee to work for his or her personal interest [7]. The main goal of such leadership is to come to an agreement on a series of actions that meet the separate and immediate aims of both the leader and the followers. Transactional leadership is accompanied by features like immobility, self-attraction and controlling the subordinates [9].

*Laissez-faire*: It is a subgroup of transactional style characterised by an attitude to avoid any responsibility and involvement. Known as “absence of leadership”, it is considered ineffective because it reduces the trust in supervisors and organisations [10]. It is a type of leadership in which followers are given complete freedom to make decisions in the leader’s absence. That is why it is considered the most passive type of leadership within the leadership spectrum [9].

*Servant*: This model encourages the professional growth of professionals and simultaneously promotes the improved delivery of healthcare services through a combination of interdisciplinary teamwork, shared decision making, and ethical behaviour [11]. Through empathy, listening to others, commitment to grow people and building community, along with their moral core, servant leaders try to help others achieve their goals and overcome challenges [7]. They share power, put the needs of others first, help individuals develop and optimize performance. They concentrate on performance planning, day-to-day coaching, and are willing to learn from others forsaking personal advancement and rewards [12].

*Resonant*: Defined as the behaviour of leaders who demonstrate a high level of emotional intelligence. They are in tune with the emotions of those around them, use empathy and manage their own emotions effectively to build strong, trusting relationships and create a climate of optimism that inspires commitment [13]. Resonant leaders coach, develop, inspire, and include others even in the case of adversity, using their emotional intelligence. They create an environment where others are highly engaged, making them willing and able to contribute with their full potential [6,14].

*Passive-avoidant*: Characterised by a leader who avoids taking responsibility and confronting others. Employees perceive the lack of control over the environment resulting from the absence of clear directives. Organisations with this type of leader have high staff turnover and low employees retention [15]. They tend to react only after problems have become serious to take corrective action, and often avoid making any decisions at all [6].

*Authentic*: Characterised by a leader with an honest and direct approach. The key elements are: self-awareness, internalised moral perspective, balanced processing, and relational transparency [16]. This model is a leader’s non-authoritarian, ethical, and transparent behaviour pattern. It strives for trusting, symmetrical, and close leader–follower relationships and promotes the open sharing of information and consideration of employees’ viewpoints [17].

Nurses’ job satisfaction should be a topic of paramount importance for healthcare organisations and their stakeholders as they represent the largest professional body of workers within healthcare systems. Several studies show that improving their job satisfaction should be a key objective in facing challenges related to achieving and maintaining quality standards, ensuring patient satisfaction and staff retention [18,19,20]. Although many published studies emphasise the importance of leadership, only a few have related the leadership styles to their effects on nurses’ job satisfaction. This research aimed to identify and analyse, through a systematic review of the literature, the current knowledge of the correlation between leadership styles and nurses’ job satisfaction.

## 2. Materials and Methods

A systematic review was performed following the PRISMA statement in order to summarise the existing literature about the correlation between leadership styles and nurses’ job satisfaction.

### 2.1. Search Strategy

The authors used the PICO model to describe all the components related to the identified problem and to structure the research question. PICO represents an acronym that stands for: Patient, Intervention, Comparison, and Outcome. These four components are the essential elements of the research question in Evidence-Based Practice for the construction of the question for the bibliographic search of evidence [21,22].

In this study, the research question was composed by PIO, in detail: (P) Nurses employed in hospital contexts; (I) use of different leadership styles by nurse coordinators, managers, and leaders; (O) effects on nurses’ satisfaction regarding their job. In order to answer the research question, Pubmed, CINAHL, and Embase databases were searched using the following string: (nurs* OR personnel OR staff OR patient OR healthcare professional OR employee) AND (leader*) AND (impact OR safety OR satisfaction OR empowerment OR performance OR attitude OR outcome) AND (hospital OR secondary care OR department OR division OR directorate OR ward OR service OR unit). The search was limited to “title/abstract” and “humans” in PubMed while no limits were set in CINHAL and Embase. No filters have been set regarding the year of publication, or regarding the type of studies.

All the resulting records published up to 22 October 2019 were screened by two independent researchers (MRC and EC). Articles were selected by first reading the title and, if deemed suitable, the abstract before finally reading the full text. Eventual uncertainties, regarding article inclusion, were overcome by discussion amongst the research members.

### 2.2. Inclusion/Exclusion Criteria

Only studies published in English and Italian, related to hospital settings, nursing focused, primary studies that have investigated a correlation between leadership styles and nurses’ job satisfaction and with availability of full text were included. The following were set as exclusion criteria: commentary study designs; articles examining leadership styles but not referring to nurses; that described effects on the leader and not on personnel; and articles that analysed effects on healthcare workers other than job satisfaction.

### 2.3. Data Synthesis

Data were entered into a spread sheet on Microsoft^®^ Excel and collated in a table which, for each article, specified the author(s), year of publication, country of origin, study design, sample, methods, results, and limitations of the study.

### 2.4. Quality Appraisal

The quality assessment was performed with the Quality Assessment Tool for Observational Cohort and Cross-Sectional Studies [23]. This scale consists of 14 questions with three possible answers: ‘yes’, ‘no’, and ‘other (cannot determine, not applicable, not reported)’. The tool evaluates the internal validity of a study, considering the risk of potential bias. After the evaluation of each criteria, the reviewer judges each study to be of ‘good’, ‘fair’, or ‘poor’ quality. A study is rated ‘good’ if the study is thought to have the least risk of bias; ‘fair’ if susceptible to some bias, which is not sufficient to invalidate the results; and ‘poor’, when there is significant risk of bias. Since the tool did not provide a clear definition of the method for the evaluation of the studies, we rated the quality of the studies based on the calculation of the tertiles of the number of items on the quality scale. The score was attributed based on the number of “yes” present and was assigned “poor” for scores falling in the lowest tertile, “fair” in the intermediate tertile, and “good” in the highest tertile.

Quality was independently evaluated by two researchers (MRC and EC) with disagreements discussed until consensus was reached.

## 3. Results

**Figure 1:**
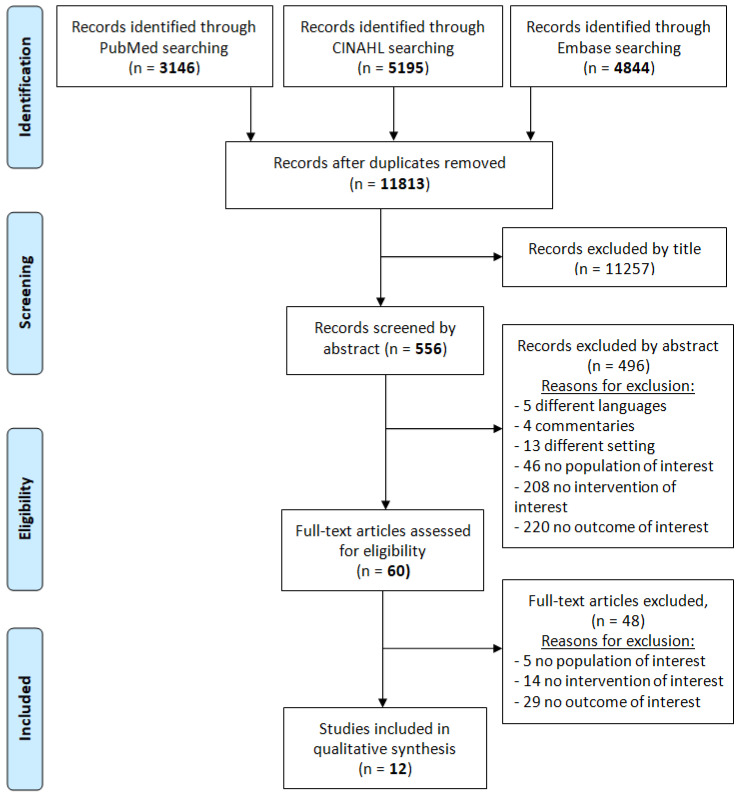
PRISMA Flowchart of articles selection.

The initial search yielded 3146 records on PubMed, 5195 on CINAHL, and 4844 on Embase. After removal of duplicates, the final records were 11,813. The selection by title reduced the eligible articles to 556 and further evaluation by abstract brought the total to 60 full texts. The aim of the research was to select only primary studies that have investigated the specific correlation between leadership styles and nurses’ job satisfaction. This contributed to the significant reduction in the number of records. In fact, only 12 studies, satisfying the stated inclusion/exclusion criteria, met the precise constraints of the research question and were eligible for the final qualitative synthesis. The process of identification, selection, and exclusion of the articles is shown in Figure 1.

Of the 12 selected studies, two (17%) were from PubMed, whilst the remaining 10 (83%) were from CINAHL. The studies included were published between 1995 and 2017 with 92% of them published within the last 8 years. Three quarters of the studies were journal articles and the remaining were dissertations [24,25,26]. All the studies could be classified as cross-sectional. In 83% of the cases, questionnaires were the tool used to evaluate job satisfaction in relation to managers’ leadership styles. Half of the studies were from North America (four from USA and two from Canada), two from Saudi Arabia, one from China, one from Ethiopia, one from Italy, and one from Jordan.

The sample size was from 83 [25] to 1216 participants [27], though in most of the studies (42%) the sample size was between 200 and 308. The Multifactor Leadership Questionnaire (MLQ) [24,25,28,29,30,31,32,33] was used in 8 out of 12 studies (67%). Other studies (33%) used the Job Satisfaction Survey (JSS) either alone [26,34] or in combination with MLQ [28,32].

Within the selected studies, there were seven leadership styles analysed: authentic, laissez-faire, passive-avoidant, resonant, servant, transactional, and transformational. Eight articles analysed more than one leadership style (from two up to four), whilst four analysed just the effects of one specific style: authentic [34], resonant [27], servant [26], and transformational leadership [35]. The transformational and the transactional leadership styles were the most frequently evaluated, appearing in 75% and 67% of the studies, respectively, followed by passive-avoidant (25%) and laissez-faire (25%).

Varying leadership styles were found to have positive, negative, or equivocal effects on nurses’ job satisfaction. Results are shown in Table 1.

Of the selected studies, 88% showed a positive or negative statistically significant correlation between leadership style and nurses’ job satisfaction. The transformational style had the greatest positive correlation in 9 out of 9 of cases. Also, authentic, resonant, and servant styles demonstrated positive correlation in 1 out of 1 case each. On the other hand, passive-avoidant and laissez-faire styles showed a negative correlation with job satisfaction in all cases (1 out of 1 each). Only in the transactional leadership style the relationship with job satisfaction was equivocal. In fact, in 3 out of 8 cases (37%) no significant correlation was found, while in 1 out of 8 cases (13%) there was a negative correlation. A positive association with this style, instead, has been found in 4 out of 8 of the studies (50%).

Details about year of publication, country, study design, sample size, methods, results, and limitations of the selected articles are shown in Table 2.

Regarding quality assessment results, all studies were found to be of fair quality. Details about quality assessment are shown in Table 3.

## 4. Discussion

In line with the findings of other studies in the literature [36,37], this review shows that, regardless of the sample, the working environment, the country or the style adopted, there is a significant correlation between leadership styles and job satisfaction. In fact, out of 12 studies included, only three of them [24,25,33] did not show any correlation between leadership style (only the transactional) and nurses’ job satisfaction. All the other studies included found significant positive or negative correlations between the two variables analysed.

Typical behaviours of servant leadership (humility, effective communication, and commitment to the professional growth of employees) show strong relation between job satisfaction and staff engagement [26]. Similarly, authentic leadership has shown, albeit only in one study, positive correlation with job satisfaction. This is in accordance with the findings of other studies [38,39].

Regarding the resonant leadership, it emerged that the ability to empower employees has both direct and indirect influence on nurses’ job satisfaction. In this specific study, the behaviour with the greatest impact and most appreciated by nursing staff was the promotion and support of teamwork by the leader [27]. This underscores the importance of ensuring staff feel part of a cohesive team, fostering an ethos of commitment and sharing of common goals.

Our results also show that leaders who adopt a transformational style, promote greater job satisfaction among nursing staff than those who adopt a transactional style. This is in line with previous studies that suggested that an open bidirectional approach to communication affects employees’ job satisfaction [40].

In most cases, transformational leaders spend time teaching and coaching nurses, focus on developing and enhancing their strengths, provide advices for their professional and personal development, treat subordinates as individuals, and listen to their concerns and doubts. When this type of style is adopted, nurses are more efficient and put more effort into achieving set goals. This finding parallels those of several other studies that have also demonstrated that adoption of a transformational leadership style has the greatest impact on healthcare system performance metrics [41,42].

The Multifactorial Leadership Model states that employees tend to be attracted by leaders who show an enthusiastic and optimistic nature and who know how to make long-term plans. The results of this literature review confirm the efficacy of this leadership model.

Just as resonant leadership has been strongly associated with promoting empowerment, so too has transformational leadership. This has a significant role in furthering employees’ sense of self-efficiency, which in turn helps promote job satisfaction.

Laissez-faire and passive-avoidant styles, in the management and coordination of personnel, are the least effective leadership styles. This research highlights the significant negative relationship between the professional satisfaction of staff and these leadership styles. This confirms the findings of previous studies in the literature [15,43,44], despite not all of them being conducted in the nursing context. The reason for this may be the fact that in both of these styles, nurses are placed under pressure to achieve set goals, without receiving guidance and practical or emotional support. These types of leadership styles do not provide clear directions and therefore employees have to set their own goals, targets, and decision-making processes. Staff may feel insecure or unattended to because they do not have consistent manager attention. Just as the laissez-faire and passive-avoidant styles are associated with a negative impact on employee performance measures, so too does the adoption of the autocratic leadership approach. Too much direction or too little communication from a manager, in fact, may negatively impact staff, leaving them feeling unmotivated and neglected.

Our research reveals that, in some cases, the transactional leadership style has a negative influence on nurses’ job satisfaction. This is line with other studies that found that the transactional leadership style is the weakest predictor of professional satisfaction [45,46]. On the contrary, in four of the selected studies there was a positive correlation between transactional leadership style and job satisfaction [28,29,30,31]. This was associated with the “Contingent Reward”, characterised by the opportunity to receive promotion and career advancement in recognition of good performance or achieved goals.

One recent study showed that the Contingent Reward, subtype of the transactional style, had characteristics common to the transformational style [47].

The reason for this could be found in the fact that professional motivation is connected to the satisfaction of deep needs such as the need of having recognised personal qualities and skills. This has a direct impact on the commitment to daily activities and on the intent to stay within the organisation.

Our research shows how more than one leadership style has positive correlation with job satisfaction. This met the authors’ aim of having the widest possible overview on the topic, beyond the identification of the “perfect” leadership style.

Different leadership styles exist because people are, by nature, different in their traits, characteristics, and communication abilities. It is therefore important for a leader to understand when a particular style must be demonstrated or avoided.

Moreover, the results reveal that most studies analysed more than one leadership style at the same time. This reflects the need to produce research which, by investigating on a broad spectrum, allows light to be shed on a topic that is still largely empirically based and with little robust scientific evidence. The fact that more than one style of leadership has led to positive outcomes amongst healthcare staff, in terms of job satisfaction, is proof that different cultures, contexts, and individuals require styles and approaches that vary over time, according to circumstances.

## 5. Conclusions

In conclusion, looking at the relation found between leadership styles and job satisfaction, we can say that nursing leaders are indispensable in creating positive work environments that retain an empowered and motivated workforce. Positive and supportive leadership styles can improve nurses’ job satisfaction, organisational commitment, and intent to stay in their position while simultaneously reducing emotional exhaustion [48].

The studies analysed in this review have revealed that transformational leadership has a significant positive correlation with levels of nursing job satisfaction. This means that transformational leaders, through their inspiring and motivating behaviour, can induce changes in the psychological states of members working within organisations.

Some of the studies analysed [27,34] have also shown that the adoption of resonant and authentic leadership styles might be decisive in improving job satisfaction through the development and strengthening of nurses’ sense of empowerment. These results suggest that leaders who focus on transparency, self-awareness, and promotion of a “work ethic” are able to empathise with their subordinates by recognising and understanding their concerns, needs and desires. Nurses who experience this type of environment longitudinally develop more confidence in their abilities and perform more effectively [49].

Furthermore, this study confirmed that perceived respect plays a key role in influencing nurses’ professional satisfaction.

Staff involvement during decision making gives them the opportunity to express personal points of view and increases a sense of mutual esteem and teamwork within the group. Institutions should promote the use of a two-way communication process and highlight the need to strengthen mutual trust between leaders and staff.

The results of this study offer a starting point for researchers, professionals, and leaders in the healthcare context to understand the benefits of adopting effective leadership styles.

The skills required for personnel management and coordination by leaders and their importance for creating successful organisations have been a literature topic for over 30 years. Despite this, much can still be investigated by future studies for the production of quantitative data generalisable to a wider range of contexts. Understanding the ideal, rather than perfect, characteristics of an effective leader should guide the process of recruiting and training personnel with management and coordination roles.

Within healthcare organisations, leadership plays a key role in providing effective and efficient care and results in positive outcomes for professionals, patients, and the work environment. It is therefore necessary to identify and fill the current gaps in leadership skills, in order to positively affect health professionals’ job satisfaction and subsequently improve healthcare quality indicators. Understanding the different effects of leadership styles allows recognition of the ways in which they impact employees, and secondly, it allows greater organisational achievement through discerning when a particular leadership style benefits or diminishes organisational goals.

The systematic review relied on a relatively limited number of eligible studies. Moreover, none of the studies were of good quality. Indeed, all of them were cross-sectional studies and therefore susceptible to bias because of the study design. This was reflected in the results of the methodological assessment, which led to the labelling of all the studies as “FAIR”. However, although no studies were rated as “GOOD”, even “POOR” studies were not found. Moreover, a limit that applies to our 12 studies, considered as a whole, has been that the criteria used to define leadership styles and the tools used to measure the level of nurses’ job satisfaction (although for some studies that are partly overlapping) are heterogeneous among the 12 studies. This did not allow to perform a rigorous meta-analysis.

Another bias to consider is the potential publication bias. It could be possible that studies with negative findings have not been published and that the correlations between leadership style and job satisfaction have been overemphasised. However, since the authors considered not only studies published in peer reviewed journals, but also dissertations, the potential publication bias may have been balanced. A thorough process of quality appraisal, based on the independent review by two authors and the use of previously validated quality appraisal tools, aided in ensuring that results were given appropriate consideration and weight and assisted the authors to determine where high quality research may have been lacking.

## Figures and Tables

**Table 1:** Correlation between leadership styles and job satisfaction.

Leadership Style	Positive Correlation	No Correlation	Negative Correlation	Total Number of Studies
Transformational	Abdelhafiz, 2016	-	-	9
	Alshahrani, 2016			
	Abualrub, 2012			
	Bormann, 2011			
	Despres, 2011			
	Medley, 1995			
	Morsiani, 2017			
	Nebiat, 2013			
	Wang, 2012			
Transactional	Abdelhafiz, 2016	Bormann, 2011 Despres, 2011 Medley, 1995	Abualrub, 2012	8
	Alshahrani, 2016			
	Morsiani, 2017			
	Nebiat, 2013			
Laissez-faire	-	-	Abdelhafiz, 2016	3
			Morsiani, 2017	
			Nebiat, 2013	
Passive-avoidant	-	-	Abdelhafiz, 2016	3
			Bormann, 2011	
			Despres, 2011	
Authentic	Wong, 2013	-	-	1
Resonant	Bawafaa, 2015	-	-	1
Servant	Mitterer, 2017	-	-	1

**Table 2:** Analysis of the studies included.

Authors, Country	Study Design, Sample Size	Methods	Results	Limitations
**Articles**				
Medley F, Larochelle D, (1995) USA	Cross-sectional *n* = 122 nurses	- Multifactor Leadership Questionnaire (MLQ); - Index of Work Satisfaction (IWS).	Positive correlation between Transformational Leadership and job satisfaction (r = 0.401; *p* < 0.001); No correlation with Transactional Leadership style (r = 0.047; *p* < 0.001)	- Quality assessed as “FAIR”
Abualrub RF, Alghamdi MG (2012) Saudi Arabia	Cross-sectional *n* = 308 nurses	- Multifactor Leadership Questionnaire (MLQ-5X); - Job Satisfaction Survey (JSS); - Statistical Package for Social Sciences (SPSS) 17.0 version.	Positive correlation between Transformational Leadership and job satisfaction (r = 0.45; *p* < 0.01); Negative correlation with Transactional Leadership (r = −0.14; *p* < 0.01)	- Convenience sample selected from 6 hospitals (results not generalisable); - Data collected after recalling participants (reporting bias possibility) - Quality assessed as “FAIR”
Wang X, Chontawan R, Nantsupawat R, (2012) China	Cross-sectional *n* = 238 nurses	- Leadership Practice Inventory (LPI); - Job Satisfaction Scale; - Statistical Package for Social Sciences (SPSS) 13.0 version.	Positive correlation between Transformational Leadership and job satisfaction (r = 0.556; *p* < 0.001)	- Quality assessed as “FAIR”
Nebiat N, Asresash, D, (2013) Ethiopia	Cross-sectional *n* = 175 nurses	- Multifactor Leadership Questionnaire (MLQ-5X); - Minnesota Satisfaction Questionnaire (MSQ); - Statistical Package for Social Sciences (SPSS) 16.0 version.	Positive correlation with Transformational (r = 0.51; *p* < 0.01) and Transactional Leadership style (β = 0.45, *p* < 0.01); Negative correlation with Laissez-faire style (r = −0.19; *p* < 0.05).	- Quality assessed as “FAIR”
Wong CA, Laschinger H, (2013) Canada	Cross-sectional *n* = 280 nurses	- Authentic Leadership Questionnaire; - Global Job Satisfaction Survey; - Statistical Package for Social Sciences (SPSS) 19.0 version.	Positive correlation between Authentic Leadership and job satisfaction (β = 0.16; *p* < 0.01)	- The cross-sectional design limits the explanations of causal effects of covariation of the variables and of the proposed theoretical associations; - Use of self-report measures does not allow to exclude variation probability - Quality assessed as “FAIR”
Bawafaa E, Wong CA, Laschinger H, (2015) Canada	Cross-sectional *n* = 1216 nurses	- Resonant Leadership Scale; - General Satisfaction Subscale; - Statistical Package for Social Sciences (SPSS) 20.0 version.	Positive correlation between Resonant Leadership and job satisfaction (r = 0.43; *p* < 0.001)	- The cross-sectional design limits the explanations of causal effects of covariation of the variables and of the proposed theoretical associations; - Low answer rate to the survey (low generalisability of the results) - Quality assessed as “FAIR”
Abdelhafiz IM, Alloubani AM, Almatari M (2016) Jordan	Cross-sectional *n* = 200 nurses	- Multifactor Leadership Questionnaire (MLQ-5X); - Job Satisfaction Tool.	Positive correlation of job satisfaction with both Transformational Leadership (r = 0.374; *p* < 0.001) and Transactional style (r = 0.391; *p* < 0.001); Negative correlation with Passive-avoidant (r = −0.240; *p* < 0.001) and with Laissez-faire styles (r = −0.225; *p* < 0.001).	- Quality assessed as “FAIR”
Alshahrani FMM, Baig LA (2016) Saudi Arabia	Cross-sectional *n* = 89 nurses	- Multifactor Leadership Questionnaire (MLQ-5X); - Job Satisfaction Survey (JSS); - Statistical Package for Social Sciences (SPSS) 20.0 version.	Positive correlation between Transformational Leadership and job satisfaction (r = 0.78; *p* < 0.01); Positive correlation between transactional leadership and job satisfaction (r = 0.50; *p* < 0.01)	- Quality assessed as “FAIR”
Morsiani G, Bagnasco A, Sasso L, (2017) Italy	Cross-sectional *n* = 87 nurses	- Multifactor Leadership Questionnaire (MLQ-5X); - STATA 12.0 version.	Positive correlation between Transformational Leadership and job satisfaction (r = 0.71; *p* < 0.01); Positive correlation with Transactional style (r = 0.55; *p* < 0.01); Negative correlation with Laissez-faire style (r = −0.42, *p* < 0.01)	- Convenience sample chosen from 3 hospitals (results not generalisable) - Quality assessed as “FAIR”
**Dissertations**				
Bormann LB, (2011) USA	Cross-sectional *n* = 115 nurses	- Multifactor Leadership Questionnaire (MLQ); - Job In General (JIG) Scale; - Job Descriptive Index (JDI).	Positive correlation between Transformational Leadership and job satisfaction (r = 0.296; *p* < 0.01); Negative correlation with Passive-avoidant style (r = −0.413; *p* < 0.01); No correlation with Transactional Leadership	- Data related to nurses’ perceptions rather than to real leadership behaviour; - Convenience sample chosen from non-profit hospitals (results not generalisable); - Low rate of response to the survey (low generalisability of the results) - Quality assessed as “FAIR”
Despres KK, (2011) USA	Cross-sectional *n* = 83 nurses	- Multifactor Leadership Questionnaire (MLQ-5X); - Job In General (JIG) Scale; - Job Descriptive Index (JDI); - JMP 5.0 version.	Positive correlation between Transformational Leadership and job satisfaction (r = 0.419; *p* < 0.05); Negative correlation with Passive-avoidant style (r = −0.419; *p* < 0.05); No correlation with Transactional style	- Convenience sample (results not generalisable); - Voluntary participation (non-participant bias); - Validity of the study limited to the reliability of the used tools; - Low rate of response to the survey (low generalisability of the results) - Quality assessed as “FAIR”
Mitterer DM, (2017) USA	Cross-sectional *n* = 283 nurses	- Servant Leadership Survey e Scale; - Job Satisfaction Survey and Job satisfaction Score; - Statistical Package for Social Sciences (SPSS) 23.0 version.	Positive correlation between Servant Leadership and job satisfaction (r = 0.44; *p* < 0.01)	- Self-reporting (reporting bias); - Cross-sectional design limits the explanations of causal effects and covariations of variables; - Voluntary participation (non-participant bias); - On-line survey (excludes all the people not familiar with technology) - Quality assessed as “FAIR”

**Table 3:** Quality Assessment Tool for Observational Cohort and Cross-Sectional Studies.

	Articles									Dissertations		
Criteria	Medley, 1995	Abualrub, 2012	Wang, 2012	Nebiat, 2013	Wong, 2013	Bawafaa, 2015	Abdelhafiz, 2016	Alshahrani, 2016	Morsiani, 2017	Bormann, 2011	Despres, 2011	Mitterer, 2017
1. Was the research question or objective in this paper clearly stated?	Yes	Yes	Yes	Yes	Yes	Yes	Yes	Yes	Yes	Yes	Yes	Yes
2. Was the study population clearly specified and defined?	No	Yes	Yes	Yes	Yes	Yes	Yes	Yes	Yes	Yes	Yes	Yes
3. Was the participation rate of eligible persons at least 50%?	No	Yes	Yes	Yes	No	No	Yes	Yes	Yes	No	No	No
4. Were all the subjects selected or recruited from the same or similar populations (including the same time period)? Were inclusion and exclusion criteria for being in the study prespecified and applied uniformly to all participants?	No	Yes	Yes	Yes	Yes	Yes	Yes	NS	Yes	Yes	Yes	Yes
5. Was a sample size justification, power description, or variance and effect estimates provided?	No	Yes	No	NA	No	No	Yes	Yes	No	Yes	Yes	Yes
6. For the analyses in this paper, were the exposure(s) of interest measured prior to the outcome(s) being measured?	No	No	No	No	No	No	No	No	No	No	No	No
7. Was the timeframe sufficient so that one could reasonably expect to see an association between exposure and outcome if it existed?	No	No	No	No	No	No	No	No	No	No	No	No
8. For exposures that can vary in amount or level, did the study examine different levels of the exposure as related to the outcome (e.g., categories of exposure, or exposure measured as continuous variable)?	Yes	Yes	Yes	Yes	Yes	Yes	Yes	Yes	Yes	Yes	Yes	Yes
9. Were the exposure measures (independent variables) clearly defined, valid, reliable, and implemented consistently across all study participants?	Yes	Yes	Yes	Yes	Yes	Yes	Yes	NS	No	Yes	Yes	Yes
10. Was the exposure(s) assessed more than once over time?	No	No	No	No	No	No	No	No	No	No	No	No
11. Were the outcome measures (dependent variables) clearly defined, valid, reliable, and implemented consistently across all study participants?	Yes	Yes	Yes	Yes	Yes	Yes	Yes	NS	No	Yes	Yes	Yes
12. Were the outcome assessors blinded to the exposure status of participants?	Yes	Yes	NS	Yes	Yes	Yes	No	NS	Yes	No	No	Yes
13. Was loss to follow-up after baseline 20% or less?	NA	NA	NA	NA	NA	NA	NA	NA	NA	NA	NA	NA
14. Were key potential confounding variables measured and adjusted statistically for their impact on the relationship between exposure(s) and outcome(s)?	Yes	Yes	No	No	No	No	No	Yes	No	No	No	Yes
**Evaluation**	**Fair**	**Fair**	**Fair**	**Fair**	**Fair**	**Fair**	**Fair**	**Fair**	**Fair**	**Fair**	**Fair**	**Fair**
